# Co-expression of Myoepithelial and Melanocytic Features in Carcinoma Ex Pleomorphic Adenoma

**DOI:** 10.1007/s12105-021-01299-4

**Published:** 2021-02-16

**Authors:** Costantino Ricci, Federico Chiarucci, Francesca Ambrosi, Tiziana Balbi, Barbara Corti, Ottavio Piccin, Ernesto Pasquini, Maria Pia Foschini

**Affiliations:** 1 Surgical Pathology Unit, Maggiore Hospital, Largo Nigrisoli 2, 40133 Bologna, BO Italy; 2 Department of Experimental, Diagnostic and Specialty Medicine (DIMES), University of Bologna, Bologna, Italy; 3 Section of Anatomic Pathology, Department of Biomedical and Neuromotor Sciences, Bellaria Hospital, University of Bologna, Bologna, Italy; 4 Surgical Pathology Unit, Sant’Orsola-Malpighi Hospital, Bologna, Italy; 5 Otolaryngology Unit, Head and Neck Surgery, Sant’Orsola-Malpighi Hospital, Bologna, Italy; 6 UOC ORL, Surgical Department, Bellaria Hospital, University of Bologna, Bologna, Italy

**Keywords:** Carcinoma, Carcinoma ex pleomorphic adenoma, Pleomorphic adenoma, Melanocytic markers, Parotid gland, Melanoma, Melanoma metastasis

## Abstract

The presence of melanin pigment and melanocytic markers expression have been rarely reported in salivary gland tumors. Herein, two cases of carcinoma arising in pleomorphic adenoma of the parotid gland and showing diffuse expression of myoepithelial and melanocytic markers are described. The clinical-pathological clues useful in the differential diagnosis with melanoma are discussed. In addition, a review of the pertinent literature is also proposed, discussing the pathologic mechanisms potentially involved in this phenomenon.

**Supplementary Information:**

The online version of this article (10.1007/s12105-021-01299-4) contains supplementary material, which is available to authorized users.

## Introduction

Multi-lineage differentiation can be observed in salivary glands carcinomas, especially in myoepithelial cell carcinoma [1]. At the best of our knowledge, only rare cases of salivary glands tumors with abundant melanin pigment and/or expression of multiple melanocytic markers (S-100, SOX10, HMB45, MART-1 and MITF) have been reported in the literature [2–11]. Several pathogenetic theories have been proposed to justify this aberrant phenotype, which could lead to a challenging differential diagnosis with metastatic and primary malignant melanoma (MM) [2–13]. Herein, we report two cases of carcinoma ex pleomorphic adenoma (PA) of the parotid gland with diffuse and strong co-expression of multiple melanocytic and myoepithelial markers. The clinical-histological features potentially helpful in the differential diagnosis with MM and the pathogenetic theories proposed to clarify this mysterious and diagnostically challenging aspect are also discussed.

## Clinical History

### Case#1

In April 2020, a 77-years-old man was referred to our institution for a PET-FDG documented a hyperaccumulation in the right parotid gland (SUVmax = 29), highly suspicious for malignancy. The patient had a previous history of MM of the left leg surgically removed in November 2018 (histotype: superficial spreading MM; pathological stage: pT2b N1a; molecular analysis: *BRAF* and *NRAS* wild type). An ultrasound-guided biopsy was performed and showed an atypical spindle-cell proliferation positive for S-100 and SOX10. Based on melanoma history and the immunohistochemical features of the salivary gland lesion, a diagnosis of “suspicious for MM metastasis” was rendered, and the patient underwent total parotidectomy. Presently, six months after surgery, the patient is alive with no evidence of disease.

### Case#2

In August 2015, a 63-years-old man underwent radiological work-up for the rapid onset of a left parotid gland swelling with facial nerve palsy. CT scan showed a 22 × 19 mm nodular lesion of the left parotid gland with infiltration of the facial nerve and suspicious for carcinoma; besides, multiple nodular lesions, radiologically suspicious for metastasis, were detected in the liver and both lungs. Extensive dermatological examination did not show a skin primary melanoma. A subtotal left parotidectomy with homolateral upper omohyoid lymphadenectomy was performed. At 4 months follow-up, a CT scan showed a size increase of the lungs and liver lesions; repeated second dermatologic examination did not reveal skin melanoma. The patient died of widespread metastatic disease seven months later.

## Pathologic Findings

### Case#1 (Fig. 1, Supplementary Material 1 and 2)

**Fig. 1:**
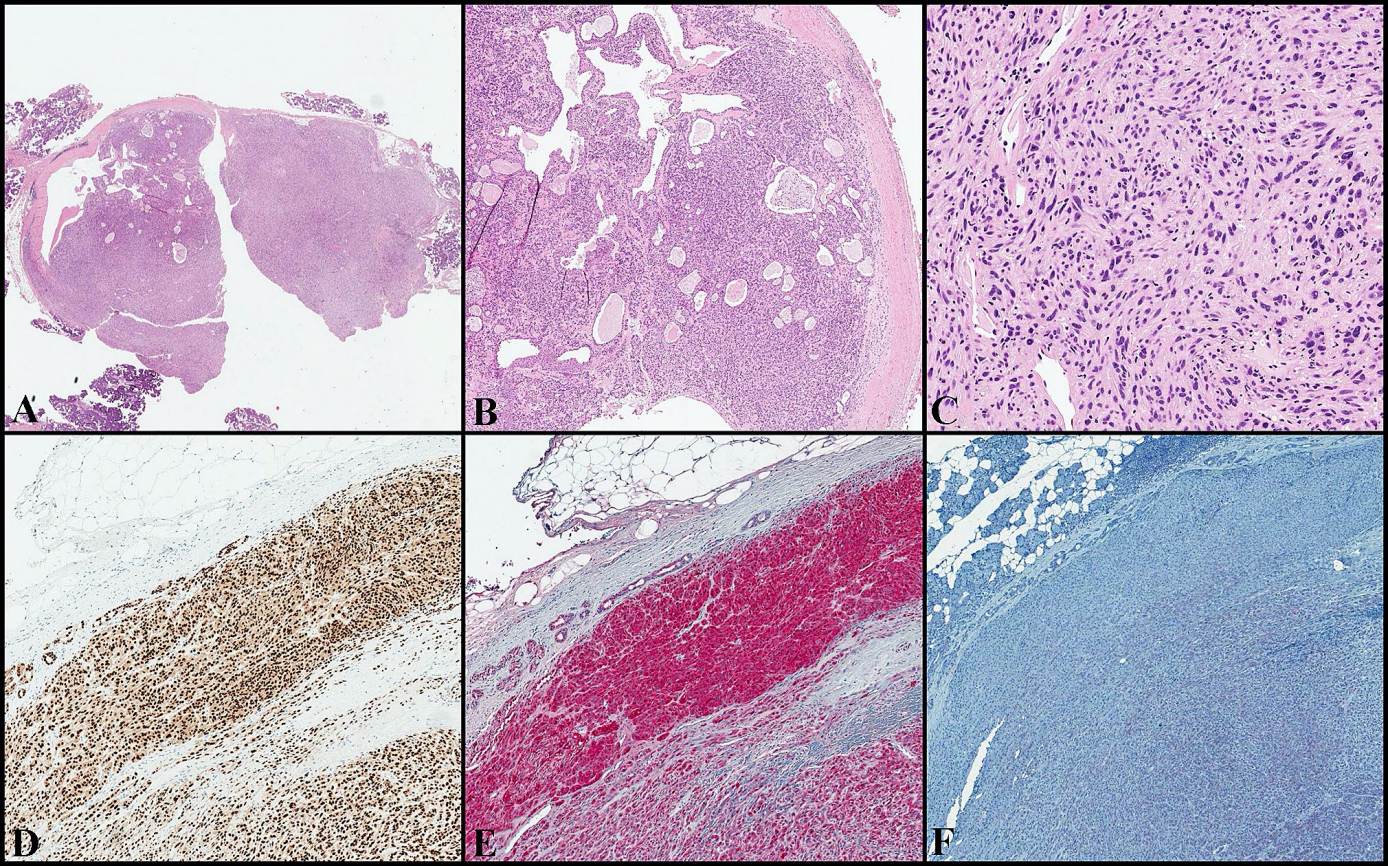
(Case#1). The C component merged with the PA but showed infiltration of the capsule and extension into the surrounding salivary gland parenchyma (**a** and **b**; H&E, original magnification × 10 and × 50). It showed a predominance of monotonous, mildly atypical and intermediated-size spindle cells, intermixed with scattered epithelioid and multinucleated ones (**c**; H&E, original magnification × 200). The neoplastic cells are positive for SOX10 (**d** original magnification × 150), S-100 (**e** original magnification × 150) and negative for MITF (**f** original magnification × 150). C: carcinoma; PA: pleomorphic adenoma; H&E: hematoxylin and eosin; SOX10: SRY-related HMG-box 10; S-100: protein S-100; MITF: Microphthalmia transcription factor

On histology, the nodule showed infiltrating margins and fascicular growth pattern. The neoplastic cells were predominantly spindle, intermixed with scattered epithelioid and multinucleated cells. Atypical mitoses were rare, in the absence of necrosis, lymph-vascular invasion and perineural infiltration. No melanin pigment was observed. The carcinomatous component gradually merged with the PA and the PA’s capsule was diffusely infiltrated.

#### Immunohistochemistry

The neoplastic cells showed diffuse and strong positivity for myoepithelial markers (SMA, desmin, p63, S-100) and low and high molecular weight cytokeratins (CK MNF116, CK CAM 5.2, CK7 and CK 34βe12). In addition, the neoplastic cells showed intense immunoreactivity for melanocytic markers as HMB45 and SOX10, while MITF and MART-1 were negative. Negativity was also observed for BRAF V600E, synaptophysin, chromogranin, CD117, DOG-1, CD45. The proliferative index (evaluated with Ki67 in the hot-spot areas) was 10%.

The slides of the skin MM (superficial spreading histotype, with a predominant nested growth pattern and composed of epithelioid and spindle cells) were reviewed by two pathologists (BC and CR) and compared with the salivary gland lesion. The two lesions resulted different in morphology (fascicular growth pattern with only spindle cells) and immunohistochemical profile, as the skin MM lacked cytokeratin and myoepithelial marker expression.

### Case#2 (Fig. 2, Supplementary Material 3)

**Fig. 2:**
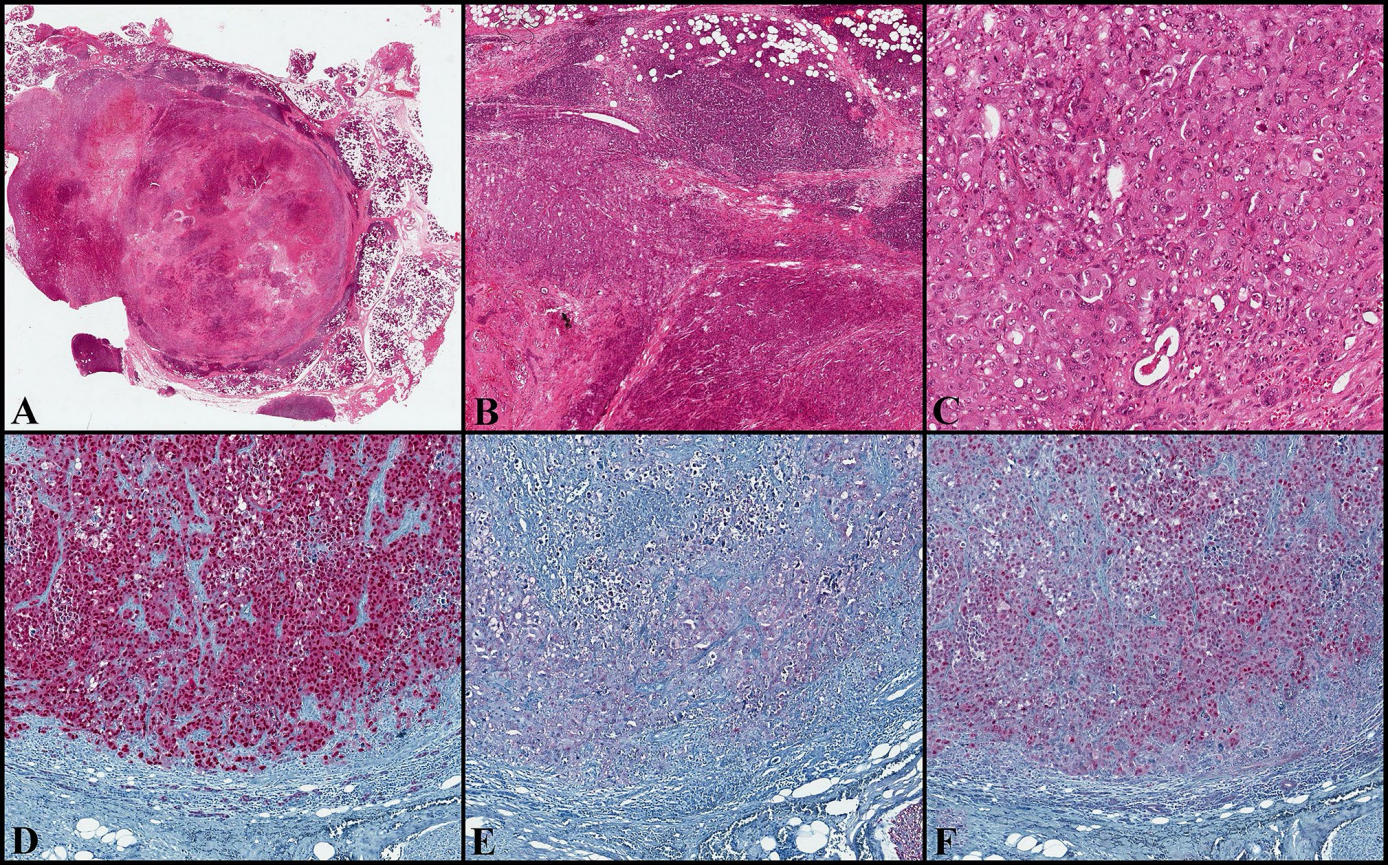
(Case#2). Pattern-less proliferation with only focally and peripherally located ductal/cribriform appearance, displaying permeative and infiltrative margins and a hyaline and focally myxoid nodular area suggestive for a pre-existing PA (**a** and **b**; H&E, original magnification × 10 and × 50). It showed a mixture of highly pleomorphic epithelioid, spindle and giant cells, with numerous atypical mitoses (**c**; H&E, original magnification × 200). The neoplastic cells are positive for SOX10 (**d** original magnification × 200), S-100 (scattered positive cells) (**e** original magnification × 200) and MITF (**f** original magnification × 200). PA: pleomorphic adenoma; H&E: hematoxylin and eosin; SOX10: SRY-related HMG-box 10; S-100: protein S-100; MITF: Microphthalmia transcription factor

On histological examination, the salivary gland parenchyma was diffusely infiltrated by a diffuse, pattern-less proliferation of neoplastic cells, displaying permeative and infiltrative margins. It was composed of a mixture of highly pleomorphic, epithelioid, spindle and giant neoplastic cells. Neoplastic cells displayed evident nucleoli and pseudo-nuclear inclusions. Atypical mitoses were numerous; large areas of necrosis, lymph-vascular invasion and perineural infiltration were present; no melanin pigment was observed. On close examination, a hyaline and myxoid nodular area suggestive for a pre-existing PA was detected in the central area of the tumor. None of the examined lymph nodes (total number: 35) showed metastatic deposits.

#### Immunohistochemistry

Focal positivity for myoepithelial (SMA, desmin, p63 and S-100) and epithelial (low molecular weight cytokeratins) markers was present. Melanocytic markers as SOX10 and MITF showed a diffuse and strong positivity, while HMB45 and MART-1 were focally present. All the remaining markers (cytokeratin 7, high molecular weight cytokeratins, BRAF V600E, synaptophysin, chromogranin, CD117, DOG-1, and CD45) resulted negative. The proliferative index, evaluated with Ki67 reached 20% in the hot-spot areas.

In both cases, a final diagnosis of carcinoma ex PA with myoepithelial/melanocytic overlapping differentiation was finally rendered. Clinical-pathological data of the two cases are summarized in Table 1; all antibodies applied in the study are listed in Supplementary Material 4.

**Table 1:** Clinical-pathological data of Cases #1 and #2

	Case #1	Case #2
Sex, Age	M, 77	M, 63
Clinical signs and symptoms	No (follow-up for melanoma)	Rapid onset of a left parotid gland swelling with facial nerve palsy
Melanoma history	Yes	No
Tumor size, major axis	15 mm	22 mm
Presence of metastasis	No	Yes (liver and lungs)
Radiological investigations	PET-FDG: hyperaccumulation in the right parotid gland (SUV max = 29);	CT total-body: nodular lesion of the parotid gland with infiltration of the facial nerve gland and multiple modular lesions in the liver and lungs suspicious for metastasis
	US: mass of the parotid gland	
Incisional biopsy	Yes (atypical spindle-cell proliferation positive for S-100 and SOX10) with a diagnosis of “suspicious for melanoma metastasis”	No
Surgical treatments	Total parotidectomy	Subtotal parotidectomy with homolateral upper omohyoid lymphadenectomy
Follow-up (months, outcome)	6, NED	7, DOD
Histological pattern	Spindle cells proliferation with fascicular growth pattern;	Pattern-less proliferation with focal and peripheral located ductal/cribriform appearance
	Pre-existing PA;	Pre-existing PA in the central area of the lesion
	Rrare atypical mitoses;	Numerous atypical mitoses
	No necrosis, lymph-vascular invasion and perineural infiltration;	Necrosis, lymph-vascular invasion and perineural infiltration;
	No melanin pigment	No melanin pigments
		Reactive lymph nodes (total number: 35)
Cytological features	Moderately atypical spindle cells with scattered epithelioid and multinucleated ones	Highly pleomorphic epithelioid, spindle and giant cells
Myoepithelial markers	SMA:+;	SMA: + (patchy);
	Desmin:+;	Desmin: + (patchy);
	S-100:+;	S-100: + (patchy);
Cytokeratin	MNF116, CAM 5.2,	MNF116, CAM 5.2: + (focal);
	7, 34βe12: + (diffuse);	7, 34βe12: -;
Melanocytic markers	SOX10, HMB45: + (diffuse);	SOX10, MITF: + (diffuse);
	MITF, MART-1: -;	HMB45, MART-1: + (focal);
BRAF V600E	–	–
Ki67 index	10%	20%

*M* male, *PET-FDG* (18)F-fluorodeoxyglucose positron emission tomography, *US* ultrasound sonography, *CT* computer tomography, *PA* pleomorphic adenoma, *NED* no evidence of disease, *DOD* dead of disease, *SMA* smooth muscle actin, *S-100* protein S-100, *SOX10* SRY-related HMG-box 10, *HMB45* Human Melanoma Black 45; *MITF* Microphthalmia transcription factor, *MART-1* Melanoma Antigen Recognized by T cells 1, *BRAF V600E* v-raf Murine Sarcoma Viral Oncogene Homolog B1 (valine at residue 600 replaced by glutamic acid), *KI67 index* proliferation index/MIB1

## Discussion

The presented two cases of carcinoma ex PA showed immunohistochemical expression of melanocytic markers, mimicking MM and potentially representing a diagnostic challenge. In both cases, the histological diagnosis of myoepithelial/melanocytic overlapping differentiation in carcinoma ex PA was reached after complete surgical resection, based on the histological features of the entire lesions and the complete immunohistochemical profile. Besides, BRAF V600E was negative in both cases.

The presence of epithelial/myoepithelial markers allowed to exclude both metastatic and exceptionally rare primary MM of the salivary glands [12, 13]. The most challenging aspect of the presented two cases and subject of numerous pathogenetic speculations is the widespread expression of multiple melanocytic markers, as well as the potential presence of melanin pigment in salivary gland tumors [2–11]. Salivary glands tumors can be “colonized” by the melanocytes resident in the salivary glands [2–8]. Melanocytes present in human oral mucosa and salivary glands of healthy patients can be activated and move to the adjacent tumor, injecting melanosomes in the neoplastic cells, similarly to what occurs with keratinocytes in the epidermis [2–6]. Melanocytic colonization can justify the aberrant expression of some melanocytic markers (HMB45 and MART-1) by salivary gland tumors [8–11]. Nevertheless, the present two cases did not show melanin pigment and/or cells with a clear-cut melanocytic morphology (large and dendritic cells, with an appearance similar to melanocytes found in the basal layer of the normal skin), but only an immunohistochemical melanocytic differentiation. In addition, positivity for markers related to melanosomes (MART-1 and HMB45) was associated with positivity for markers of melanocytic differentiation as SOX10 and MITF; furthermore, the positivity was so strong and widespread throughout the entire lesion to be difficultly explainable just by melanosomes presence in the neoplastic cells. The alternative pathogenetic theory is a myoepithelial/melanocytic overlapping differentiation, justified by a common neural crest origin and proved by numerous shared immunohistochemical markers such as S-100 [7]. Myoepithelial cells are well-known for the potential acquisition of divergent phenotypes, as epithelioid, spindle, adipocytic, clear, plasmacytoid, squamous with or without keratin [7, 10, 11]. *Desai SS *et al*.* reported a case of adenoid cystic carcinoma with abundant melanin in the neoplastic myoepithelial cells, with no evidence of intratumoral cells resembling dendritic melanocytes [7]. The authors interpreted this phenomenon as a demonstration that myoepithelial cells can acquire a melanocytic-like phenotype [7]. As result, the melanocytic immunophenotype and the synthesis of melanin by myoepithelial cells should be considered as an alternative “phenotypic avatar” potentially shown by these plastic cells [7]. Additional proof of the myoepithelial/melanocytic overlapping differentiation is the widespread SOX10 expression observed in salivary glands [9, 10]. *Ohtomo *et al*.* reported that in normal human salivary gland tissue, SOX10 expression was observed in the nuclei of acini and both luminal and abluminal cells of intercalated ducts [10]. The same authors showed as SOX10 is expressed from the developmental stage to adulthood, associated with the presence of epithelial stem/progenitor cells from embryonic day 13.5 (E13.5) [10]. In the neoplastic counterpart, SOX10 is observed in a broad group of tumors as acinic cell carcinomas, adenoid cystic carcinomas, epithelial-myoepithelial carcinomas, myoepithelial carcinomas, basal cell adenoma and pleomorphic adenoma [9, 10]. These data suggest that a broad family of salivary glands tumors, regardless of a specific lineage, derive from neural crest stem cells (SOX-10 positive) and may display a plastic and hybrid phenotype not always clearly definable [10]. For this reason, *Ohtomo *et al*.* proposed a revolutionary classification of salivary glands neoplasia in SOX10 + tumors (originating from neural crest stem cells) and SOX10 – ones (alternative origin) [10]. Melanocytic differentiation has been reported also in metaplastic breast carcinoma and rare examples of soft tissue tumors with bi or multi-directional differentiation [14–16]. It remains to be determined whether this apparently paradoxical differentiation is the result of the transformation of a cancer stem cell (histogenesis theory) rather than a trans-melanocytic differentiation occurring as a late-step change (dedifferentiation theory) [14–16]. The cases of salivary gland tumors with melanin pigment and/or melanocytic phenotype reported in the literature are too few to obtain data on the possible prognostic impact of the myoepithelial/melanocytic overlapping differentiation [2–11]. The reported cases were different in histological subtypes and AJCC clinical-pathological stages, which does not allow to obtain easily comparable data on the possible prognostic impact of this rare and intriguing phenomenon [2–11]. Carcinoma ex PA could show a broad range of histological features and, as consequence, of clinical aggressivity and prognosis (Case#1 had a low-grade malignant component and a very short follow-up; Case#2 had a pleomorphic and high-grade malignant component and the patient died of widespread metastatic disease 7 months after the diagnosis); besides, Case#2 showed at onset multiple lesions in liver and lungs, radiologically suspicious for metastasis (IV AJCC clinical stage). As result, the present cases do not add specific prognostic information as we think that the different histology and AJCC clinical stage could justify the clinical course of the two patients, regardless of the co-expression of myoepithelial and melanocytic features. Future studies are needed to clarify if this this aberrant melanocytic phenotype could have clinical and prognostic relevance.

## Conclusions

Herein, two cases of carcinoma ex PA with myoepithelial/melanocytic overlapping differentiation of the parotid gland are presented, together with the clinical-pathological features helpful in the differential diagnosis with MM. Possible pathogenic theories justifying this curious and diagnostic challenging aspect are also discussed.

## Supplementary Information

Below is the link to the Supplementary Information.

Supplementary Material 1 (Case#1). PA and C components were both positive for p63 (A, original magnification x50), CK CAM 5.2 (B, original magnification x50), CK MNF 116 (C, original magnification x50) and negative for BRAF V600E (D, original magnification x50).PA: pleomorphic adenoma; C: carcinoma; p63: p63 protein; CK CAM 5.2: cytokeratin CAM 5.2 (CK 7 and 8); CK MNF 116: cytokeratin MNF 116 (CK 5, 6, 8, 17 and 19); BRAF V600E: v-raf Murine Sarcoma Viral Oncogene Homolog B1 (valine at residue 600 replaced by glutamic acid). Supplementary Information 1 (TIFF 16222 kb)

Supplementary Material 2 (Case#1). The primary skin MM of superficial spreading type (A, original magnification x150; B, original magnification x150) was negative for p63 (C, original magnification x150) and positive for MART-1 with a high Ki67 index (D, original magnification x150). MM: malignant melanoma; p63: p63 protein; MART-1: Melanoma Antigen Recognized by T cells 1; Ki67: Ki67 proliferative index. Supplementary Information 2 (TIFF 14400 kb)

Supplementary Material 3 (Case#2). The C component was focally positive for p63 (A, original magnification x150), CK CAM 5.2 (B,original magnification x150), CK MNF 116 (C, original magnification x150) and negative for BRAF V600E (D, original magnification x150). C: carcinoma; p63: p63 protein; CK CAM 5.2: cytokeratin CAM 5.2 (CK 7 and 8); CK MNF 116: cytokeratin MNF 116 (CK 5, 6, 8, 17 and 19); BRAF V600E: v-raf Murine Sarcoma Viral Oncogene Homolog B1 (valine at residue 600 replaced by glutamic acid). Supplementary Information 3 (TIFF 16222 kb)

Supplementary Information 4 (DOCX 20 kb)

## Data Availability

All data generated and/or analyzed during this study are included in this published article [and its supplementary information files].
