# Supplementary figures and images for: Co-expression of Myoepithelial and Melanocytic Features in Carcinoma Ex Pleomorphic Adenoma
Source: Head Neck Pathol. 2021 Feb 16;15(4):1385–90. doi: 10.1007/s12105-021-01299-4 (PMC8633150; doi:10.1007/s12105-021-01299-4)

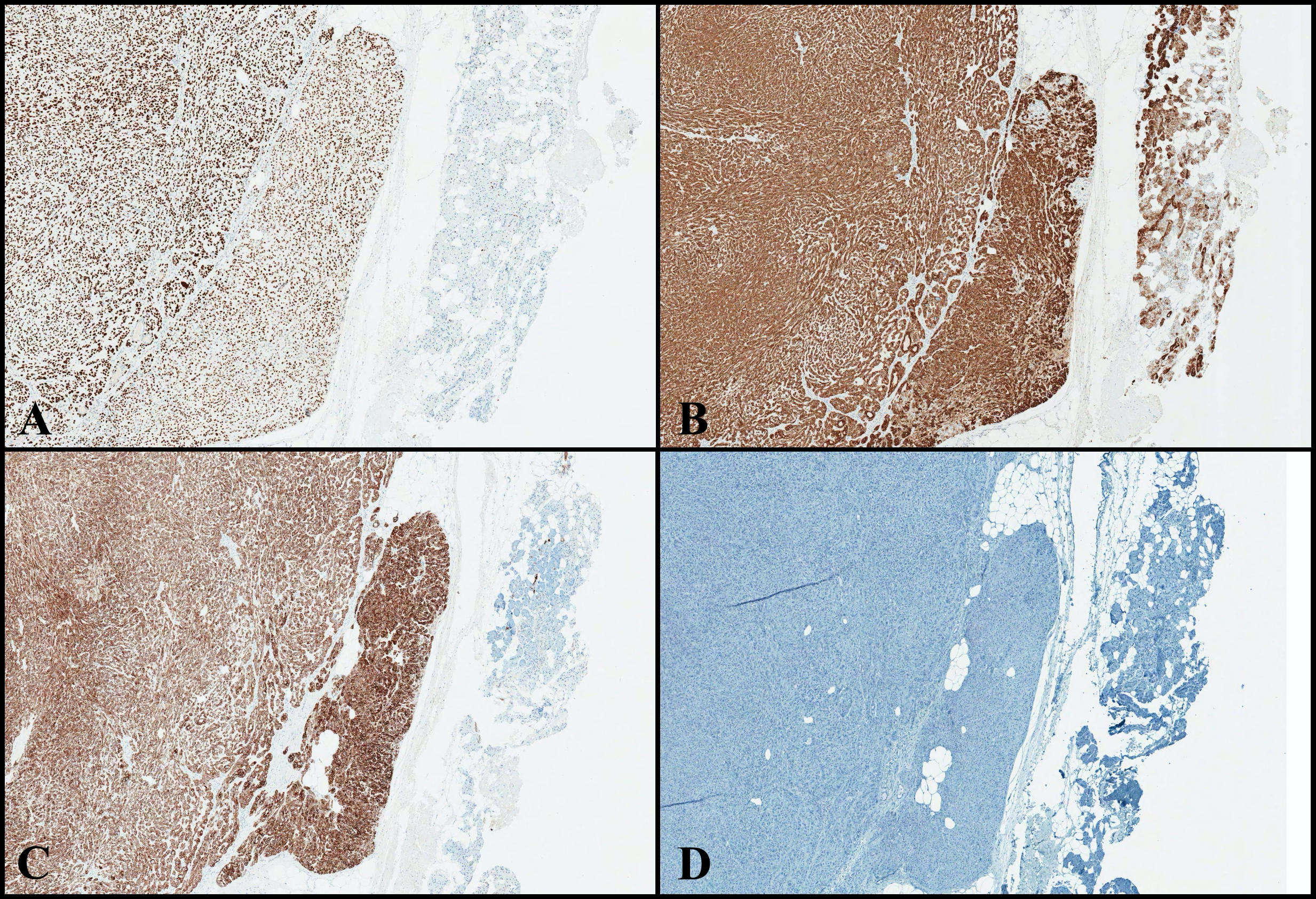

Supplement: Supplementary file 1 — Supplementary Material 1 (Case#1). PA and C components were both positive for p63 (A, original magnification x50), CK CAM 5.2 (B, original magnification x50), CK MNF 116 (C, original magnification x50) and negative for BRAF V600E (D, original magnification x50).PA: pleomorphic adenoma; C: carcinoma; p63: p63 protein; CK CAM 5.2: cytokeratin CAM 5.2 (CK 7 and 8); CK MNF 116: cytokeratin MNF 116 (CK 5, 6, 8, 17 and 19); BRAF V600E: v-raf Murine Sarcoma Viral Oncogene Homolog B1 (valine at residue 600 replaced by glutamic acid). Supplementary Information 1 (TIFF 16222 kb) [file 12105_2021_1299_MOESM1_ESM.tiff]

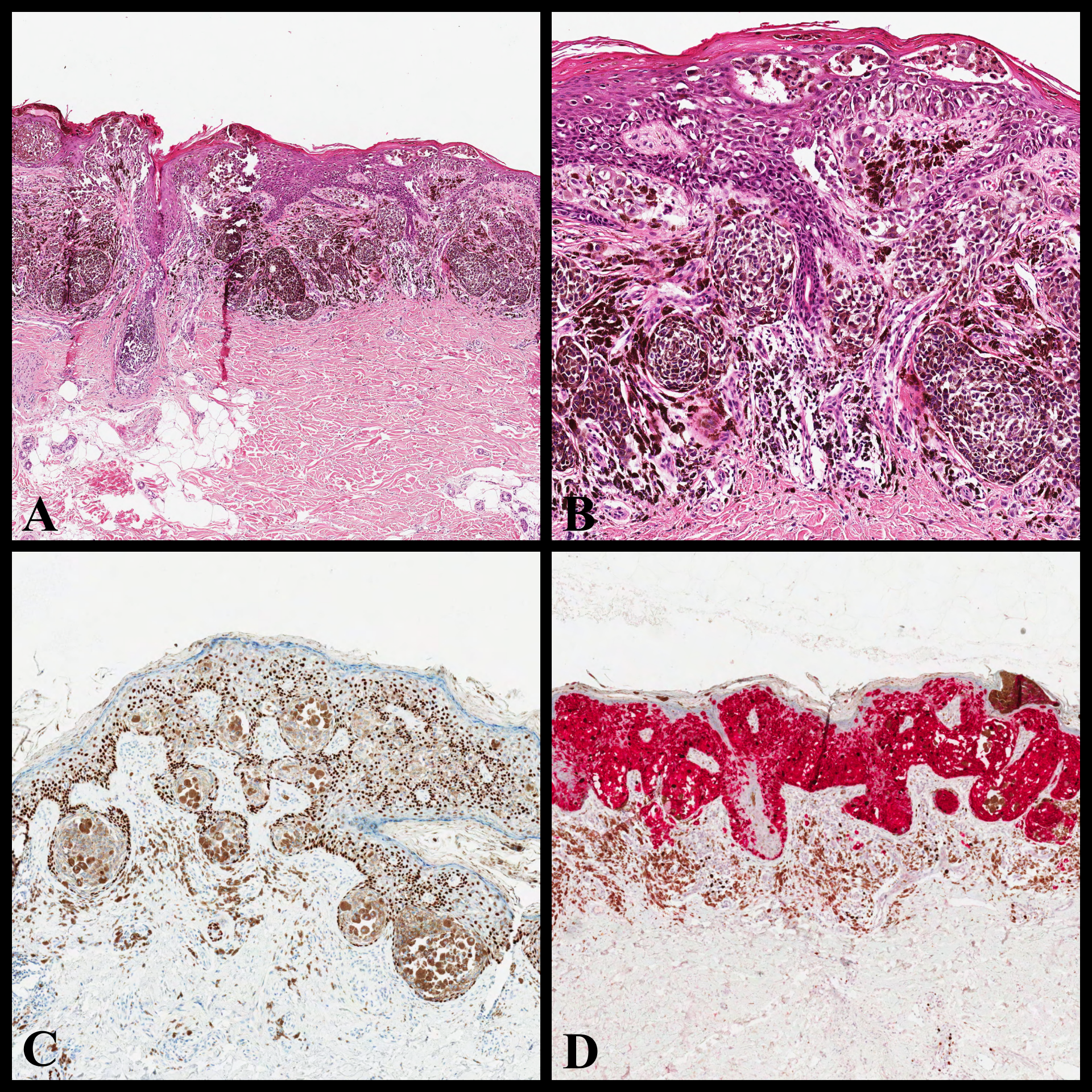

Supplement: Supplementary file 2 — Supplementary Material 2 (Case#1). The primary skin MM of superficial spreading type (A, original magnification x150; B, original magnification x150) was negative for p63 (C, original magnification x150) and positive for MART-1 with a high Ki67 index (D, original magnification x150). MM: malignant melanoma; p63: p63 protein; MART-1: Melanoma Antigen Recognized by T cells 1; Ki67: Ki67 proliferative index. Supplementary Information 2 (TIFF 14400 kb) [file 12105_2021_1299_MOESM2_ESM.tiff]

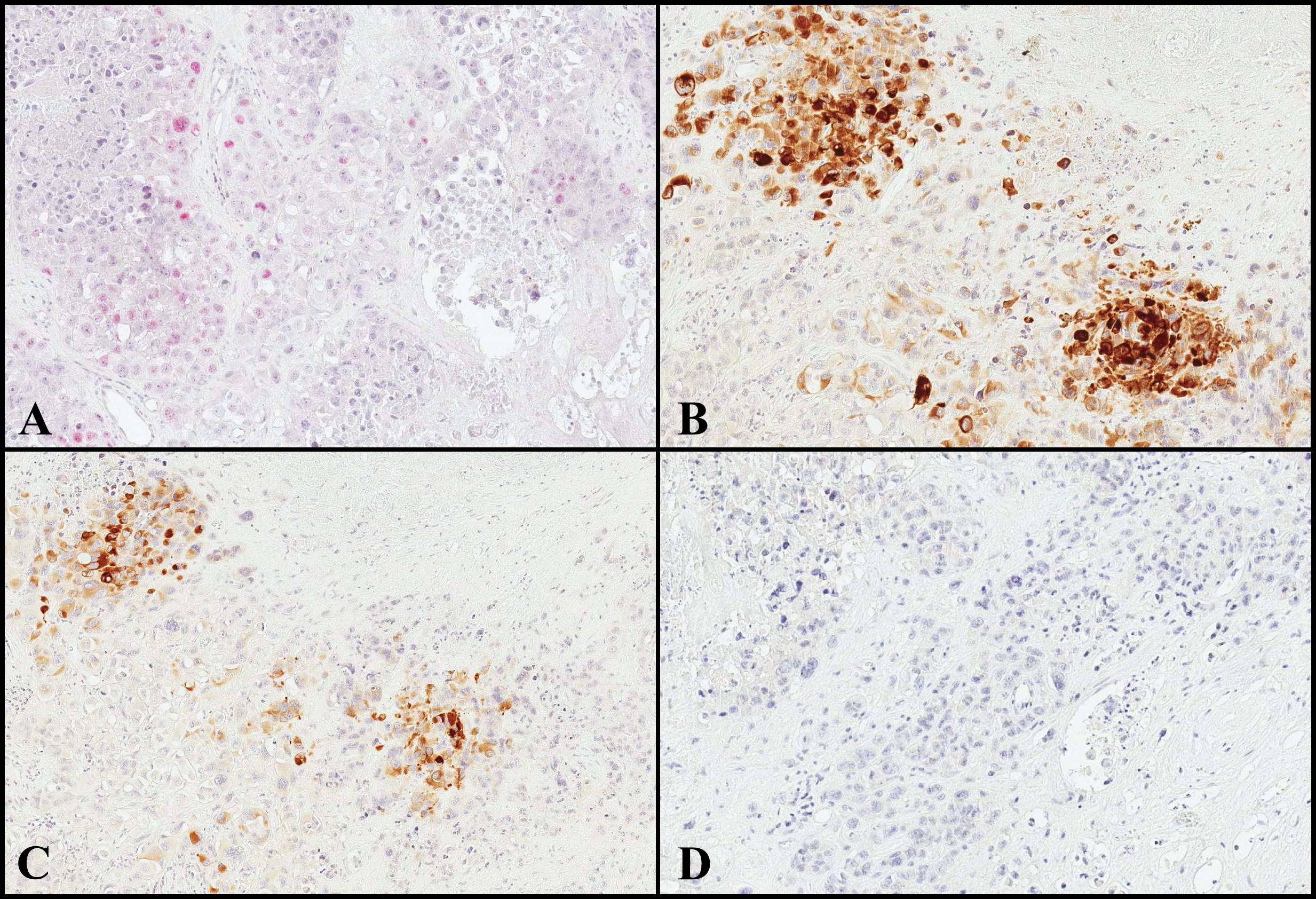

Supplement: Supplementary file 3 — Supplementary Material 3 (Case#2). The C component was focally positive for p63 (A, original magnification x150), CK CAM 5.2 (B,original magnification x150), CK MNF 116 (C, original magnification x150) and negative for BRAF V600E (D, original magnification x150). C: carcinoma; p63: p63 protein; CK CAM 5.2: cytokeratin CAM 5.2 (CK 7 and 8); CK MNF 116: cytokeratin MNF 116 (CK 5, 6, 8, 17 and 19); BRAF V600E: v-raf Murine Sarcoma Viral Oncogene Homolog B1 (valine at residue 600 replaced by glutamic acid). Supplementary Information 3 (TIFF 16222 kb) [file 12105_2021_1299_MOESM3_ESM.tiff]
