# Supplementary material for: Co-expression of Myoepithelial and Melanocytic Features in Carcinoma Ex Pleomorphic Adenoma
Source: Head Neck Pathol. 2021 Feb 16;15(4):1385–90. doi: 10.1007/s12105-021-01299-4 (PMC8633150; doi:10.1007/s12105-021-01299-4)
Supplement: Supplementary file 4 — Supplementary Information 4 (DOCX 20 kb) [file 12105_2021_1299_MOESM4_ESM.docx]

| **Antibody** | **Clone name** | **Species** | **Dilution** | **Chromogens (labelling)** |
| --- | --- | --- | --- | --- |
| **SOX-10** | SP267 | Rabbit (monoclonal) | 1:900 | DAB (Case#1) AEC (Case#2) |
| **HMB45** | HMB-45 | Mouse (monoclonal) | RTU | DAB |
| **MART-1** | Melan A | Mouse  (monoclonal) | RTU | AEC |
| **MITF** | D5 | Mouse (monoclonal) | RTU | AEC |
| **Desmin** | DE-R-11 | Mouse (monoclonal) | RTU | DAB |
| **SMA** | 1A4 | Mouse (monoclonal) | RTU | DAB |
| **S-100** | Polyclonal | Rabbit (polyclonal) | RTU | AEC |
| **p63** | 4A4 | Mouse (monoclonal) | RTU | DAB (Case#1) AEC (Case#2) |
| **BRAF V600E** | VE1 | Mouse (monoclonal) | RTU | AEC |
| **CK MNF116** | MNF 116 | Mouse (monoclonal) | RTU | DAB |
| **CK CAM 5.2** | CAM5.2 | Mouse (monoclonal) | RTU | DAB |
| **CK7** | SP52 | Rabbit (monoclonal) | RTU | DAB |
| **CK 34βe12** | 34β-E12 | Mouse (monoclonal) | RTU | DAB |
| **CD117** | c-kit | Rabbit (monoclonal) | RTU | DAB |
| **DOG-1** | SP31 | Rabbit (monoclonal) | RTU | DAB |
| **CD45** | 2B11 | Mouse (monoclonal) | RTU | DAB |
| **Ki67** | 30-9 | Rabbit  (monoclonal) | RTU | AEC |

**SOX-10**: SRY-related HMG-box 10; **HMB45**: human melanoma black 45; **MART-1**: melanoma antigen recognized by T cells 1; **MITF**: melanocyte inducing transcription factor; **SMA**: smooth muscle actin; **S-100**: S-100 protein; **p63**: p63 protein; **BRAF**: v-raf Murine Sarcoma Viral Oncogene Homolog B1 (valine at residue 600 replaced by glutamic acid); **CK MNF116**: cytokeratin MNF 116 (CK 5, 6, 8, 17 and 19); **CK CAM 5.2**: cytokeratin CAM 5.2 (CK 7 and 8); **CK 7**: cytokeratin 7; **CK** **34βe12**: cytokeratin 34βe12 (CK 1, 5, 10 and 14); **CD117**: cluster of differentiation 117/c-kit; **DOG-1**: discovered on GIST-1; **CD45**: cluster of differentiation 45; **Ki67**: proliferation index/MIB1; **RTU**: ready to use; **DAB**: 3,3’-Diaminobenzidine (brown color); **AEC**: 3-Amino-9-ethylcarbazole (red color).

**Supplementary Material 4.
Technical data of antibodies used in the study.**
